# Comprehensive bioinformatic analysis constructs a CXCL model for predicting survival and immunotherapy effectiveness in ovarian cancer

**DOI:** 10.3389/fphar.2023.1127557

**Published:** 2023-03-09

**Authors:** Shuang Li, Dawei Zou, Zhaoqian Liu

**Affiliations:** ^1^ Hunan Key Laboratory of Pharmacogenetics, Department of Clinical Pharmacology, National Clinical Research Center for Geriatric Disorders, Xiangya Hospital, Central South University, Changsha, China; ^2^ Institute of Clinical Pharmacology, Central South University, Changsha, China; ^3^ Department of Surgery, Immunobiology and Transplant Science Center, Houston Methodist Research Institute and Institute for Academic Medicine, Houston Methodist Hospital, Houston, TX, United States

**Keywords:** immunotherapy, the C-X-C motif chemokine ligands (CXCLs), tumor microenvironment, ovarian cancer, prognosis

## Abstract

**Background:** Immunotherapy has limited effectiveness in ovarian cancer (OC) patients, highlighting the need for reliable biomarkers to predict the effectiveness of these treatments. The C-X-C motif chemokine ligands (CXCLs) have been shown to be associated with survival outcomes and immunotherapy efficacy in cancer patients. In this study, we aimed to evaluate the predictive value of 16 CXCLs in OC patients.

**Methods:** We analyzed RNA-seq data from The Cancer Genome Atlas, Gene Expression Omnibus, and UCSC Xena database and conducted survival analysis. Consensus cluster analysis was used to group patients into distinct clusters based on their expression patterns. Biological pathway alterations and immune infiltration patterns were examined across these clusters using gene set variation analysis and single-sample gene set enrichment analysis. We also developed a CXCL scoring model using principal component analysis and evaluated its effectiveness in predicting immunotherapy response by assessing tumor microenvironment cell infiltration, tumor mutational burden estimation, PD-L1/CTLA4 expression, and immunophenoscore analysis (IPS).

**Results:** Most CXCL family genes were overexpressed in OC tissues compared to normal ovarian tissues. Patients were grouped into three distinct CXCL clusters based on their CXCL expression pattern. Additionally, using differentially expressed genes among the CXCL clusters, patients could also be grouped into three gene clusters. The CXCL and gene subtypes effectively predicted survival and immune cell infiltration levels for OC patients. Furthermore, patients with high CXCL scores had significantly better survival outcomes, higher levels of immune cell infiltration, higher IPS, and higher expression of PD-L1/CTLA4 than those with low CXCL scores.

**Conclusion:** The CXCL score has the potential to be a promising biomarker to guide immunotherapy in individual OC patients and predict their clinical outcomes and immunotherapy responses.

## Introduction

Ovarian cancer (OC) is estimated to be the fifth leading cause of cancer-related deaths among women in the United States in 2023 (Siegel et al., 2023). The standard treatment for OC involves radical surgery and chemotherapy (Ledermann et al., 2013), but the 5-year survival rate for OC patients remains low despite these efforts (Oronsky et al., 2017). Immunotherapy has emerged as a promising new approach for treating various types of cancer (Kraehenbuehl et al., 2022). The FDA has approved six types of immune checkpoint inhibitors (ICIs) for cancer therapy since 2011, including targeting cytotoxic T lymphocyte-associated protein 4 (CTLA-4), programmed death-1 (PD-1), and programmed death-ligand 1 (PD-L1) (Hargadon et al., 2018). Although ICIs have shown success in treating several cancers, not all patients with ovarian cancer respond to immunotherapy (Hamanishi et al., 2021; Moore et al., 2021). Therefore, it is critical and urgent to identify new and effective strategies to guide immunotherapy in OC patients, in order to improve their outcomes.

Biomarkers are essential in directing the efficacy of immunotherapy in cancer and enhancing patient outcomes. The use of specific biomarkers can help predict which patients will likely respond positively to immunotherapy, allowing for a tailored treatment plan. One significant biomarker is the expression of specific proteins, such as PD-L1, on the surface of cancer cells. High PD-L1 expression has been linked to improved responses to immunotherapy drugs that target this protein (Patel and Kurzrock, 2015). Another biomarker is the presence of immune cells, known as tumor-infiltrating lymphocytes (TILs), within the tumor tissue. High levels of TILs have also been associated with improved responses to immunotherapy (Presti et al., 2022). Moreover, the genetic composition of the tumor can also impact the response to immunotherapy. For instance, mutations in genes like TP53 have been connected with improved responses to immunotherapy in cancer (Dong et al., 2017). However, the current biomarkers do not fully explain the responses to immunotherapy in OC, and there is a pressing need for more effective biomarkers to guide immunotherapy in this patient population.

C-X-C motif chemokine ligands (CXCLs) are a group of chemical molecules that guide cell migration and are widely associated with tumor progression and response to immunotherapy (Charo and Ransohoff, 2006; Markl et al., 2022). For instance, CXCL1 has been linked to the promotion of cancer cell migration and the progression of gastric cancer (Wang et al., 2017) and breast cancer metastasis (Wang et al., 2018). On the other hand, CXCL8 is a target for solid tumor immunotherapy (Dominguez et al., 2017), and CXCL9/10 has been demonstrated to enhance the accumulation of effector T cells at the tumor site and suppress tumor growth (Karin, 2020). In ovarian cancer, high levels of CXCL1 expression have been found to promote cancer progression by inducing cell proliferation (Bolitho et al., 2010), whereas CXCL9 has been shown to potentiate anti-tumor activity and drive a positive response to anti-PD-L1 therapy (Seitz et al., 2022). Despite these findings, the systematic predictive value of CXCLs in terms of overall survival and response to immunotherapy in individual OC patients remains unclear.

Our study aimed to systematically evaluate the role of CXCLs in OC prognosis and immunotherapy. We found that most of the CXCL family genes were overexpressed in OC compared to normal tissues, and were independent predictors of patient outcomes. Based on the expression levels of CXCLs, OC patients were grouped into three distinct CXCL patterns or gene clusters, each with a distinct relationship to patient outcome and immune cell infiltration. Additionally, we developed a CXCL scoring model using principal component analysis (PCA), which accurately predicted the prognosis and immunotherapy response of individual patients with OC. Patients with high CXCL scores had improved survival, increased immune cell infiltration, and a higher sensitivity to immunotherapy.

## Materials and methods

### Data download and processing

To assess the expression of CXCLs in normal and OC tissues, we collected 88 normal ovary samples and 427 ovarian cancer samples with normalized TPM (transcripts per kilobase million) from the UCSC Xena database (https://xena.ucsc.edu/). The gene transcription data and clinical information of OC were obtained from The Cancer Genome Atlas (TCGA) (https://portal.gdc.cancer.gov/) and Gene Expression Omnibus (GEO) (https://www.ncbi.nlm.nih.gov/geo/) databases, which were merged into a TCGA-GEO matrix (totaling 758 samples) after adjusting for batch effects using the “SVA” R package. We used the “limma” R package to compare the expression levels of CXCLs between normal and OC tissues. Information on copy number and somatic mutations was also obtained from the UCSC Xena database for generating Circos plots with the “RCircos” R package and calculating the tumor mutational burden (TMB) with the “maftools” R package. Survival analysis was conducted using Cox regression analysis and Kaplan–Meier (KM) methods, statistical significance was defined as a p-value less than 0.1 for Cox regression analysis and less than 0.05 for Kaplan-Meier methods.

### Consensus cluster analysis to build clusters based on the expression of CXCLs

We used consensus cluster analysis to group the TCGA-GEO cohort based on the expression levels of the 16 CXCLs, with the help of the “ConsensusClusterPlus” R package (Wilkerson and Hayes, 2010). The analysis showed that grouping the samples into three clusters (k = 3) had the best association of intra-typical samples, a low coefficient of variation, and an adequate sample size for each cluster. The fitness of the classification was evaluated using Principal Component Analysis (PCA) (Ringner, 2008). A heatmap of the CXCL expression levels among the three CXCL clusters and their corresponding clinical features was generated using the “pheatmap” R package.

### Gene set variation analysis (GSVA) of three CXCL clusters

To understand the distinct biological pathways associated with the three CXCL clusters, we conducted gene set variation analysis (GSVA) using the “GSVA” R package (Hanzelmann et al., 2013). The “c2. cp.kegg.v2022.1. Hs.symbols” gene set was obtained from the GSEA website (https://www.gsea-msigdb.org) and used to analyze the enrichment of gene sets in each of the three CXCL clusters. This analysis aimed to provide insights into the biological processes that may contribute to the observed differences in CXCL expression between the three clusters. The top 20 enriched pathways were visualized in a heatmap, with adjusted *p*-values less than 0.05 considered significant.

### Infiltration levels of immune cells

The tumor microenvironment (TME) infiltration immune cell type was defined by Zhang et al. (2020). The relative infiltration levels of each type of immune cell in each sample were calculated using the single-sample gene set enrichment analysis (ssGSEA) (Subramanian et al., 2005). The enrichment score represented the enrichment of each type of immune cell in the sample. The correlation between the CXCL score and each type of infiltration immune cell was analyzed using the “corrplot” R package.

### Differentially expressed genes analysis among CXCL patterns

To identify the differentially expressed genes (DEGs) among the three CXCL patterns, a differentially expressed genes analysis was performed using the “limma” R package on the normalized TPM data of 758 ovarian cancer patients from the three CXCL clusters (Smyth, 2004). A significance threshold of adjusted *p*-value <0.001 was applied to filter DEGs. As a result, 3811 DEGs were identified between CXCL clusters A and B, 552 DEGs between CXCL clusters A and C, 1941 DEGs between CXCL clusters B and C, and 244 shared DEGs. The results of differentially expressed genes analysis were visualized using Venn diagrams generated by the “VennDiagram” R package. The shared DEGs were further evaluated for their potential biological functions using Gene Ontology (GO) (Ashburner et al., 2000) and Kyoto Encyclopedia of Genes and Genomes (KEGG) pathway enrichment analysis (Kanehisa and Goto, 2000). A univariate Cox regression analysis was conducted to identify shared survival related DEGs, and a significance threshold of *p* < 0.05 was applied. Based on the expression levels of shared survival related DEGs, the TCGA-GEO cohort was grouped into three gene clusters using consensus cluster analysis. The expression of shared survival related DEGs in the three gene clusters was visualized using the “pheatmap” R package, and the “limma” R package was used to analyze the expression profiles of 16 CXCLs among the three gene subtypes.

### Differences in survival among CXCL clusters, gene clusters, or CXCL score model

For survival analysis, patients with missing follow-up information were excluded. The probability of survival was compared across CXCL clusters, gene clusters, and CXCL score groups, respectively, using the “survival” and “survminer” R packages. The assessment of the survival curves was performed through the Kaplan-Meier method and log-rank tests.

### Estimating tumor mutational burden (TMB)

The tumor mutational burden (TMB) is calculated as the number of mutated bases per million bases. The simple nucleotide variations of OC patients were obtained from the TCGA database and processed using Practical Extraction and Report Language (Perl) version 5.30.0. The patients were divided into two groups, high TMB and low TMB, based on the optimal cutoff value of TMB. Survival analysis was performed to compare the prognosis between the high and low TMB groups and to assess the impact of TMB on prognosis when combined with CXCL scores.

### Immunophenoscore (IPS) analysis in the CXCL score model

Charoentong et al. introduced the Immune Prediction Score (IPS), which is used to predict a patient’s response to checkpoint blockade in cancer (Charoentong et al., 2017). The clinical data and IPS for OC patients were obtained from The Cancer Immunome Atlas (https://tcia.at/). In this study, the IPS was analyzed to evaluate the effectiveness of immunotherapy in OC patients with high and low CXCL scores.

### Construct a CXCL score model

To evaluate the predictive value of CXCLs in individual patients, we developed a CXCL score model based on the expression levels of the shared survival related DEGs among the three CXCL clusters. The CXCL score was calculated by summing the signature scores, which were extracted from the PCA as the first and second principal components (PC1 and PC2). The formula for defining the CXCL score is as follows (Ringner, 2008).

$$CXCL\;score=\sum \left({PC1}_{i}+{PC2}_{i}\right)$$

Where “i” represents the expression levels of the shared survival related DEGs among the three CXCL clusters.

### Statistical analysis

Statistical analyses were conducted using R software version 4.2.1. The Student’s t-test or Wilcoxon rank-sum test was applied to evaluate the distribution of variables. The Log-rank test or Kruskal-Wallis test was used to compare data between two or more groups, respectively. Correlations between two variables were analyzed using Pearson or Spearman correlation analysis. The “survival” R package was used to subgroup samples. Kaplan-Meier survival analysis and univariate Cox regression analysis were performed using the “survminer” package.

## Results

### The characteristics of CXCLs in OC

**FIGURE 1:**
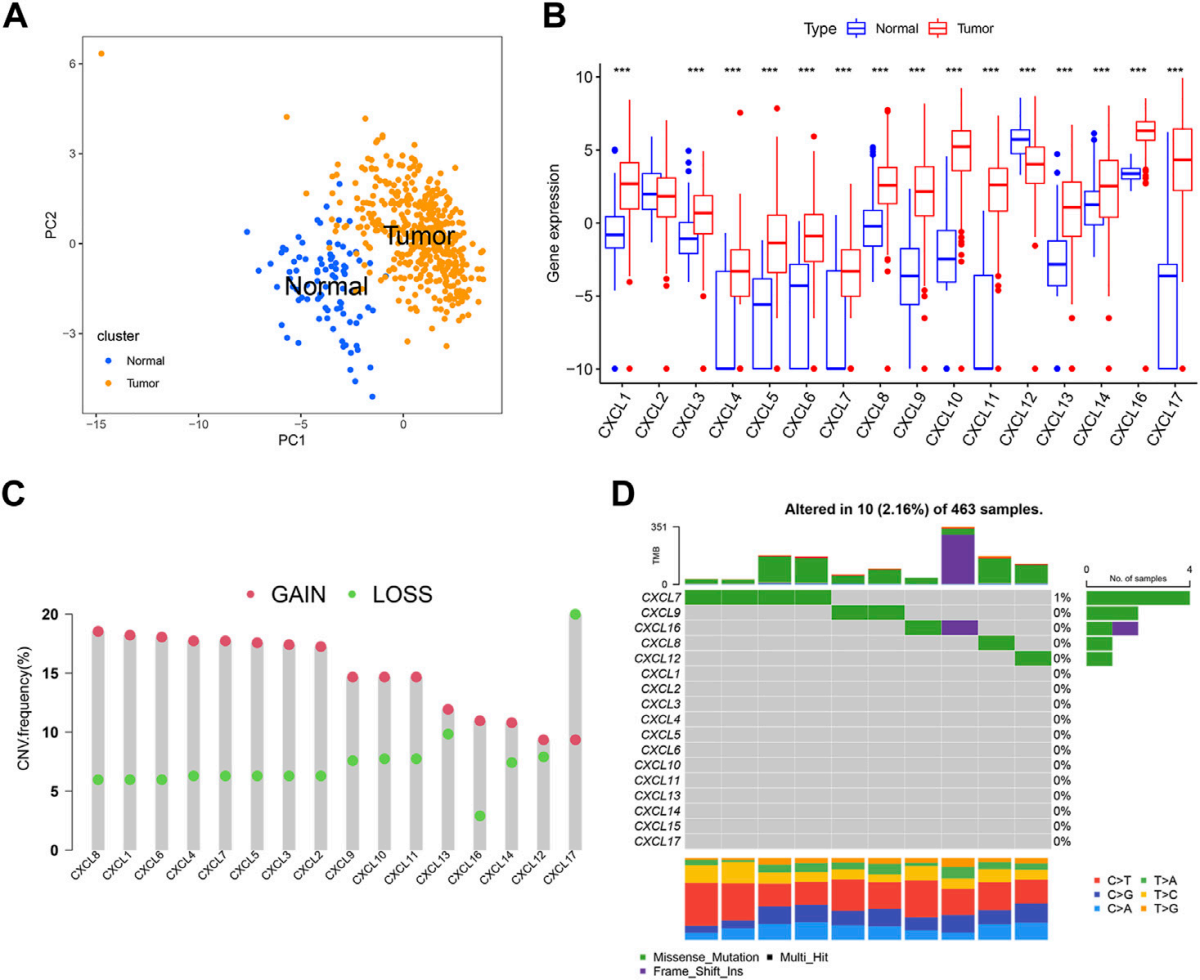
Characteristics of CXCLs in OC. **(A)** The PCA plots demonstrate a clear distinction between normal and OC tissue, with blue dots representing normal tissue and yellow dots representing tumor tissue. **(B)** The box plots depict the mRNA expression (log2 x + 1) profiles of 16 CXCLs in normal and OC tissue. ***p < 0.001. **(C)** The CNV frequency of CXCLs in TCGA-OC is illustrated by the height of the column, with red dots representing an increase in frequency and green dots representing a decrease in frequency. **(D)** The somatic mutation rate of CXCL family genes in TCGA-OC patients. The bottom bar graph represents mutation transformation.

To investigate the characteristics of CXCLs in OC, we compared the expression levels of 16 CXCL family genes between normal ovarian tissues and OC tissues using the UCSC Xena and TCGA databases. The PCA results showed that the gene expression profiles of normal and OC tissue were different (Figure 1A). Among the CXCLs, mRNA levels of CXCL1, CXCL3, CXCL4, CXCL5, CXCL6, CXCL7, CXCL8, CXCL9, CXCL10, CXCL11, CXCL13, CXCL14, and CXCL16 were upregulated in OC, while the expression of CXCL12 was significantly higher in normal ovarian tissue (Figure 1B). We also analyzed copy number variation (CNV) and somatic mutation frequency of the CXCL family genes using the TCGA-OC cohort. Figure 1C shows that, for most CXCLs (excluding CXCL17), the frequency of gain copy number was higher than that of lost copy number (Figure 1C). CXCL family genes (CXCL7, CXCL9, CXCL16, CXCL6, and CXCL12) showed somatic mutation events in 10 of 463 TCGA-OC samples, with CXCL7 exhibiting the highest mutation frequency (4/463) (Figure 1D).

### The prognostic value of CXCLs in OC

**FIGURE 2:**
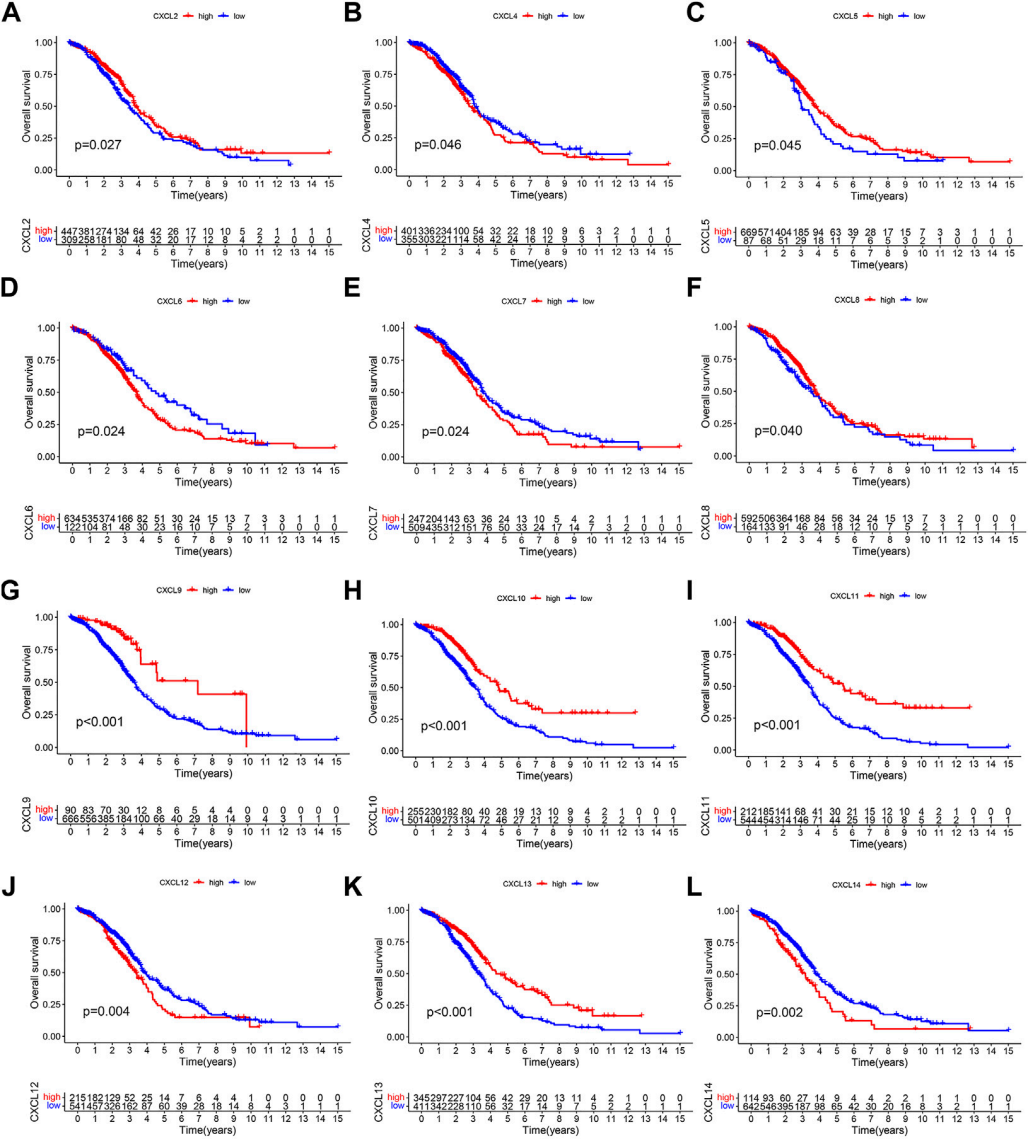
The prognostic significance of CXCLs in OC. Kaplan-Meier curve displays the difference in overall survival between patients with high and low expression levels of **(A)** CXCL2, **(B)** CXCL4, **(C)** CXCL5, **(D)** CXCL6, **(E)** CXCL7, **(F)** CXCL8, **(G)** CXCL9, **(H)** CXCL10, **(I)** CXCL11, **(J)** CXCL12, **(K)** CXCL13, and **(L)** CXCL14. All data are derived from TCGA-OC and GSE140082 datasets.

To assess the prognostic value of individual CXCL family genes in OC patients, we performed Kaplan-Meier (K-M) survival analysis and univariate Cox regression analysis using the TCGA and GSE140082 databases. The K-M curve showed that the expression levels of CXCL4, CXCL6, CXCL7, CXCL12, and CXCL14 were associated with worse survival outcomes (Figures 2B, D, E, J, L), while the expression levels of CXCL2, CXCL5, CXCL8, CXCL9, CXCL10, CXCL11, and CXCL13 were correlated with better overall survival (OS) of patients (Figures 2A, C, F–I, K). Univariate Cox regression analysis also showed that 5 CXCL family genes (CXCL9, CXCL10, CXCL11, CXCL13, and CXCL14) were single risk factors for the OS of patients (Supplementary Table S1). These results suggest that CXCL9, CXCL10, CXCL11, CXCL13, and CXCL14 could be used as potential prognostic biomarkers for OC patients.

### CXCLs expression-based subtypes in OC patients

**FIGURE 3:**
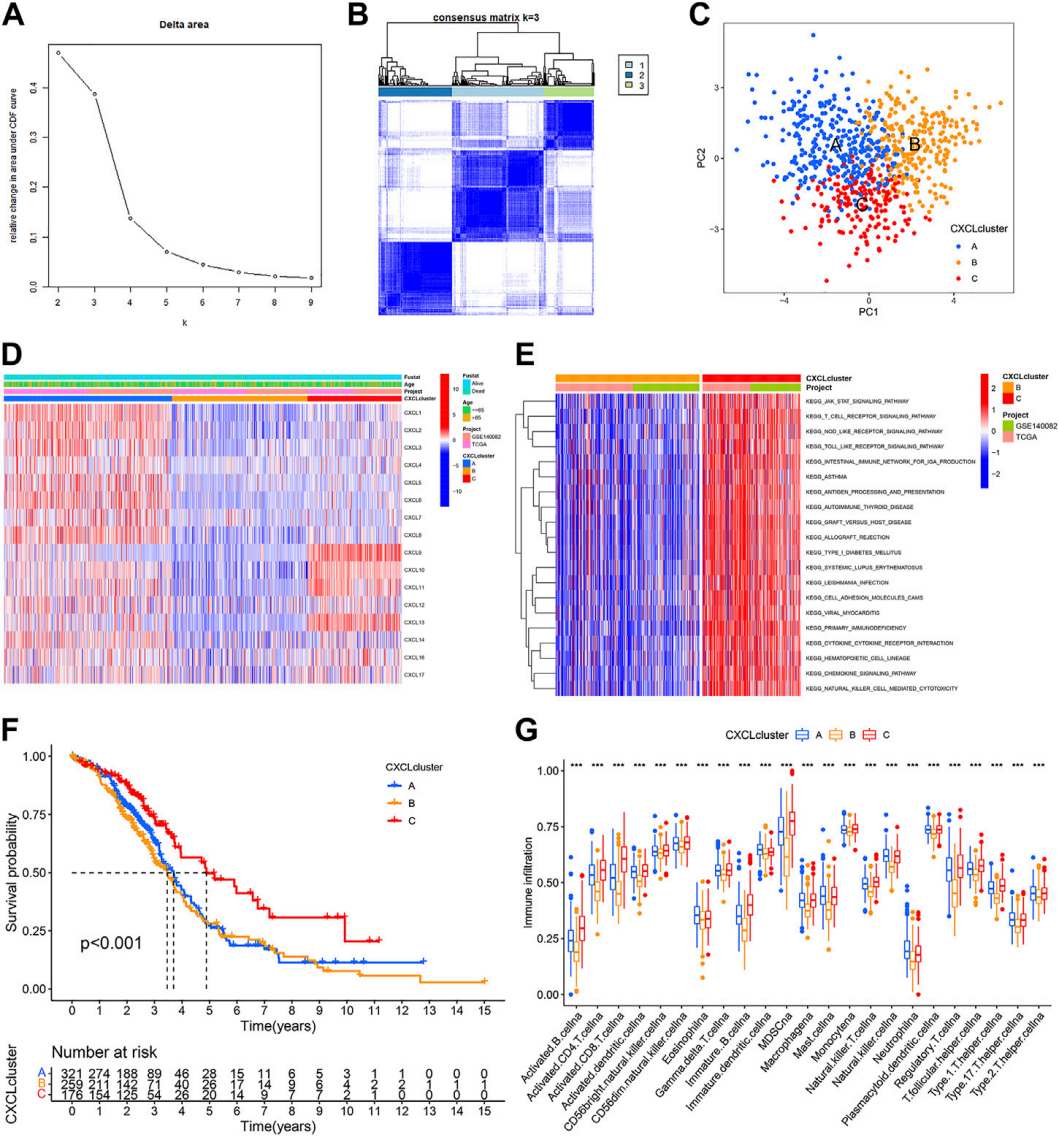
CXCLs expression-based subtypes of OC patients. **(A,B)** Consensus clustering analysis was performed based on the expression of CXCLs. The consensus cumulative distribution function (CDF) from k = 2 to 9 is shown in **(A)**, and the heatmap of the consensus matrix (k = 3) is shown in **(B)**. **(C)** PCA plots of the three CXCL subtypes. Blue dots represent CXCL cluster A, yellow dots represent CXCL cluster B, and red dots represent CXCL cluster C. **(D)** The mRNA expression of 16 CXCLs in the three subtypes is shown in a heatmap. **(E)** A heatmap shows the results of KEGG pathway enrichment analysis between CXCL clusters B and C. **(F)** The Kaplan-Meier curve displays the outcome of patients in the three CXCL subtypes. **(G)** Box plots display the level of immune cell infiltration in the three CXCL subtypes. ***p < 0.001.

To understand the role of CXCLs in OC, patients from the TCGA-OC and GSE140082 cohorts were combined and grouped into multiple patterns based on the similarity of CXCL family member expressions using consensus cluster analysis. The analysis identified three subgroups based on the lack of significant increase in the area under the cumulative distribution function (CDF) curve and the clear boundaries observed between the subgroups (Figures 3A, B). The differences among the subclasses were further evaluated using PCA, which revealed that CXCL clusters A, B, and C were significantly distinct from each other (Figure 3C). The heatmap showed that CXCL family genes were upregulated in CXCL cluster A and downregulated in CXCL cluster B (Figure 3D). In cluster C, some CXCL genes were upregulated while others were downregulated (Figure 3D).

To gain further insights into the biological differences among the three CXCL clusters, Gene Set Variation Analysis (GSVA) was performed. Results indicated that CXCL cluster C was mainly associated with immune responses, such as regulating the T cell receptor and Toll-like receptor signaling pathways (Figure 3E,Supplementary Figures S1A, B). A survival analysis was also conducted on patients in each CXCL cluster. The results showed that patients in CXCL cluster C had a better overall survival rate compared to those in CXCL cluster A and B (*p* < 0.001) (Figure 3F). Single-sample gene set enrichment analysis (ssGSEA) revealed a significantly higher infiltration of immune cells in CXCL cluster C compared to CXCL clusters A and B, which may explain the better survival outcomes of patients in CXCL cluster C compared to CXCL clusters A and B (Figure 3G). Therefore, OC patients can be successfully divided into three subtypes based on the similarity of CXCLs expression.

### Identification of three gene clusters based on the expression patterns of DEGs among three CXCL clusters

**FIGURE 4:**
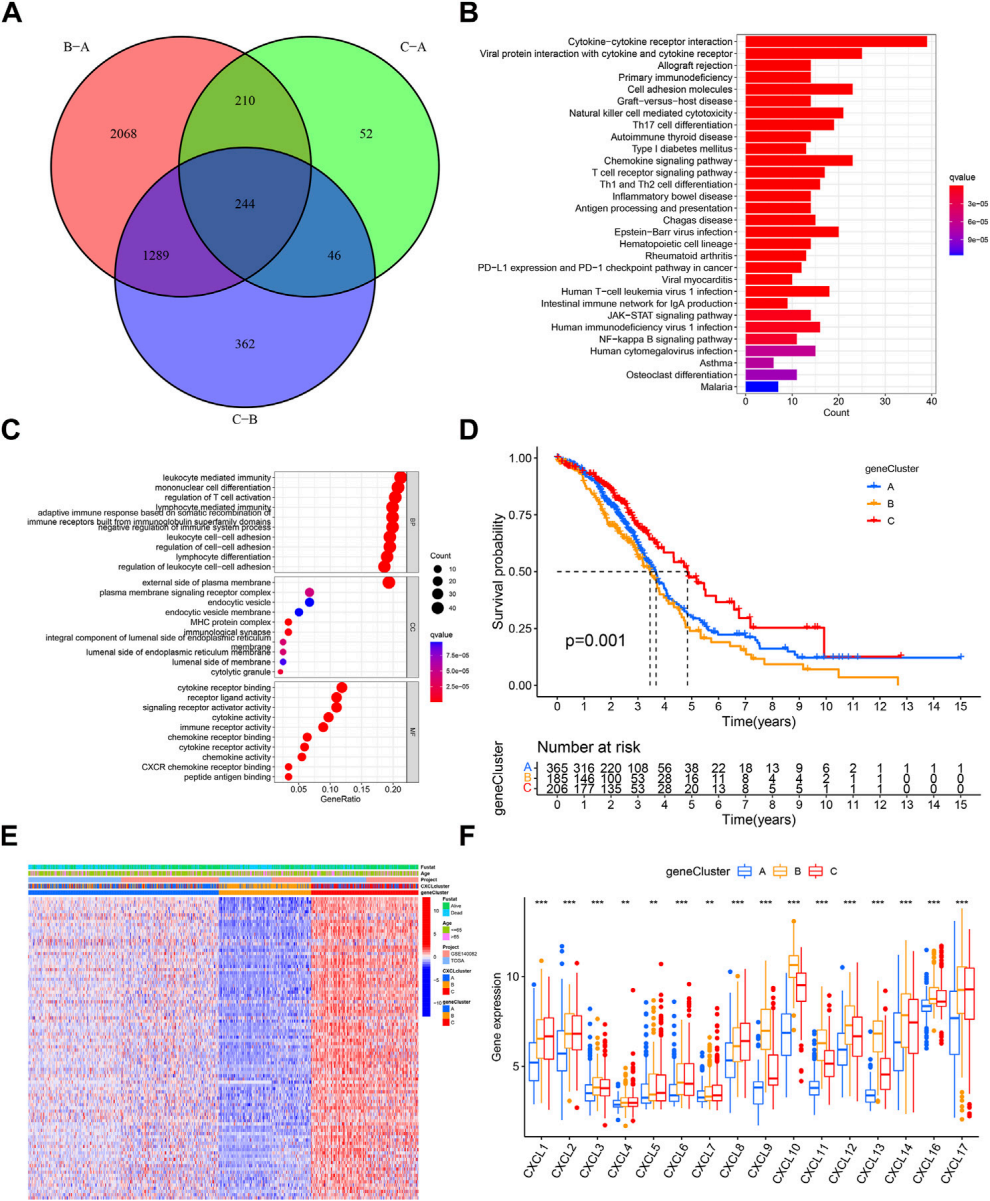
Three prognosis gene clusters in OC. **(A)** Venn diagram displays DEGs among three gene subtypes, with 244 genes identified as shared DEGs. **(B)** The top 30 enriched KEGG pathways based on the shared DEGs. **(C)** Dot plots show the top 10 GO terms in each biological process, based on the shared DEGs. **(D)** Kaplan-Meier curve displays the survival probability of patients in the three gene subtypes. **(E)** The heatmap displays the distribution of shared survival related DEGs, clinical characteristics, and the CXCL cluster in the three gene clusters. **(F)** Box plots display the expression of CXCLs in the three gene clusters. **p < 0.01, ***p < 0.001.

To further examine the biological significance of the CXCLs, 244 shared DEGs were identified across the three CXCL subtypes (Figure 4A). These shared DEGs were found to be enriched in several immune cell-related pathways, such as T cell differentiation (Figures 4B, C; Supplementary Figures 2A, B), and 94 DEGs were significantly associated with the OS of patients (Supplementary Table S2). Patients were then divided into three gene clusters based on these 94 OS-related shared DEGs (Supplementary Figures 3A, B). The Kaplan-Meier curve showed that patients in gene cluster C had a better survival rate compared to patients in gene clusters A and B (Figure 4D). The expression profile of the 94 OS-related shared DEGs along with clinical characteristics among the three gene clusters is displayed in a heatmap (Figure 4E). Additionally, we compared the expression levels of the CXCLs in the three gene clusters. As indicated by the box plot, patients in gene cluster A expressed the lowest mRNA levels of the CXCLs, while most patients in gene cluster B had higher expression levels of CXCLs than those in gene cluster C (Figure 4F). In conclusion, patients can be effectively separated into three gene clusters that can predict their overall survival.

### Construct a CXCL score model

**FIGURE 5:**
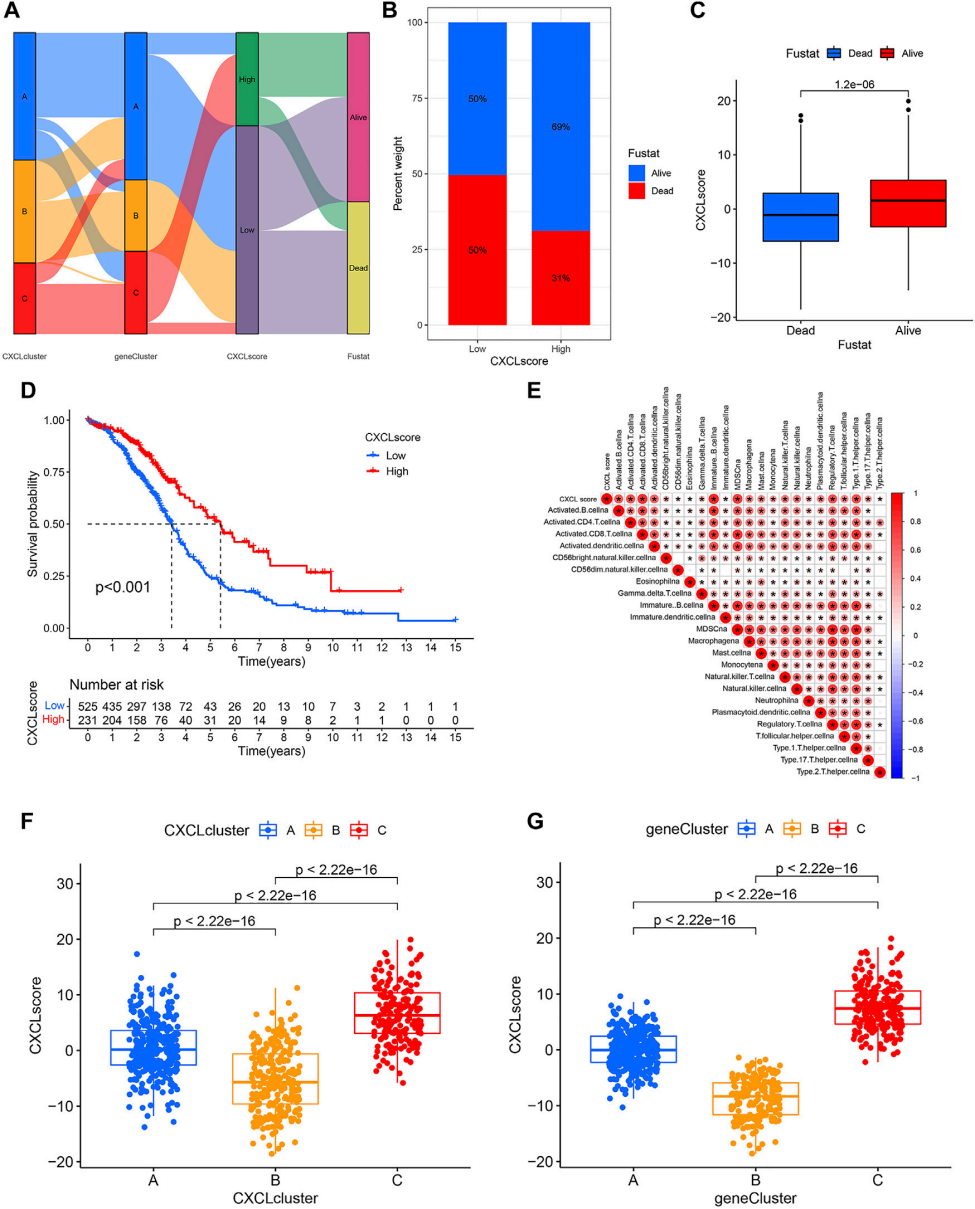
Construct a CXCL score model through PCA. **(A)**The Sankey diagram illustrates the connections between CXCL clusters, gene clusters, CXCL score patterns, and patient survival outcomes. **(B)** The distribution of patients with low or high CXCL scores who are alive or deceased. **(C)** Bar plots show the CXCL score distribution in patients with different prognoses. **(D)** Kaplan-Meier curve displays the survival probability of patients with varying CXCL scores. **(E)** The relationship between CXCL score and the level of immune cell infiltration is shown, with blue indicating negative correlations and red indicating positive correlations. **(F,G)** Bar plots illustrate the difference in CXCL score between the three CXCL clusters **(F)** and three gene clusters **(G)**, respectively, with *p* values indicated.

Due to the diversity of tumors, we utilized PCA methodology to accurately evaluate the CXCL pattern of individual OC patients, which was named the CXCL score. The attribute changes of individual patients were depicted in a Sankey diagram (Figure 5A). Next, OC patients were divided into high-score and low-score groups based on their CXCL scores, and the survival outcomes of patients in these two groups were compared. The results showed that patients with high CXCL scores had a better survival rate compared to those with low CXCL scores (Figures 5B–D). We then analyzed the correlation between CXCL score and immune cell infiltration level using Spearman correlation analysis. Our results showed that a high CXCL score was positively correlated with higher immune cell infiltration (Figure 5E). Additionally, we calculated the CXCL scores of patients in different CXCL clusters and gene clusters. Among the three CXCL patterns, patients in CXCL cluster C had the highest CXCL score, while CXCL cluster B had the lowest CXCL score (Figure 5F). In the three gene clusters, the average CXCL score of patients in gene cluster C was higher than those in gene clusters A and B (Figure 5G). Therefore, the CXCL score may serve as a potential positive biomarker for predicting the prognosis of OC patients.

### The association between CXCL score and TMB/somatic mutation rates

**FIGURE 6:**
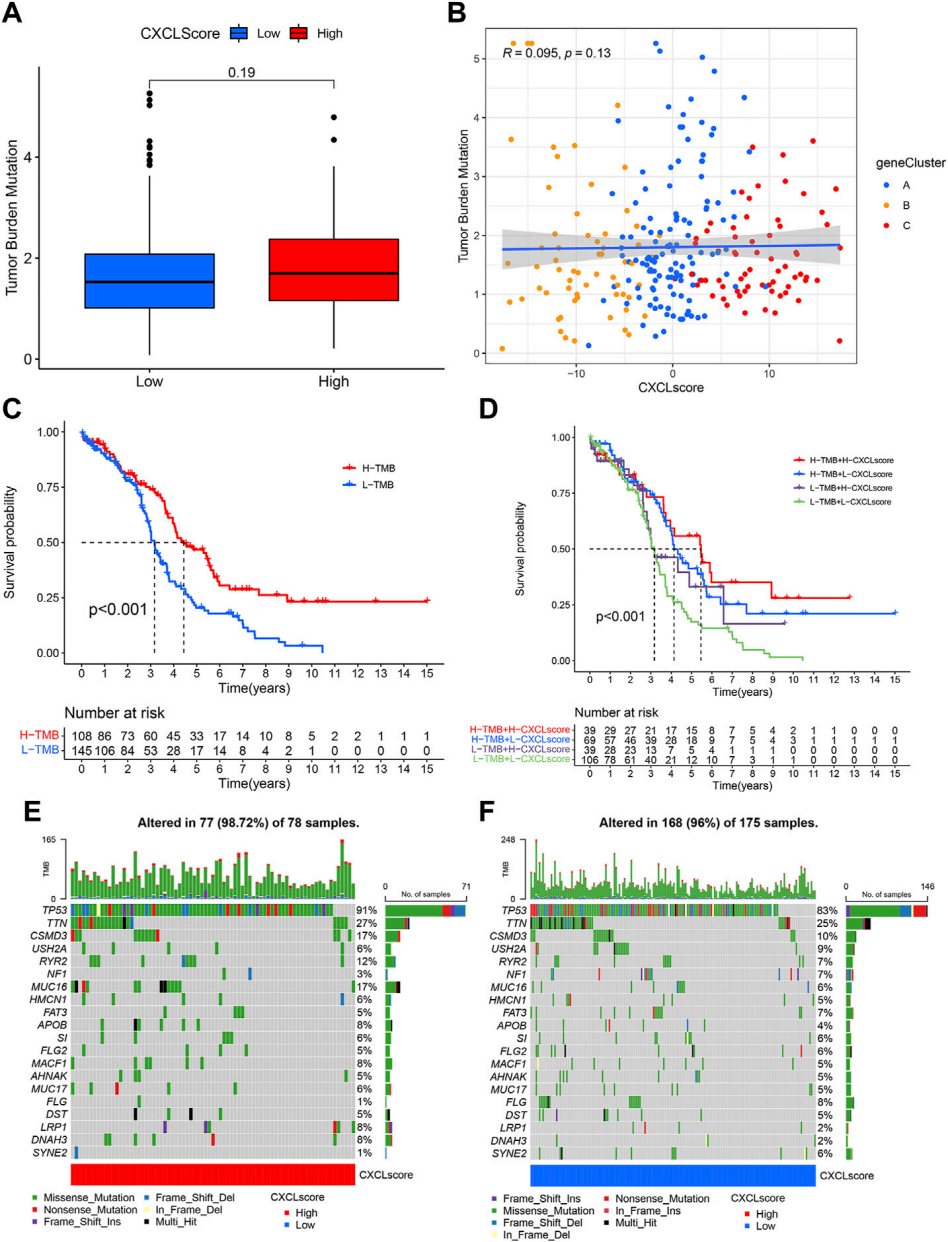
The association between CXCL score and TMB/somatic mutation rates. **(A)** Box plots show the TMB values in the high and low CXCL score groups. The *p*-value is indicated. **(B)** Scatter plots demonstrate the relationship between CXCL score, TMB, and the three gene clusters. Gene Cluster A is represented by blue dots, Gene Cluster B by yellow dots, and Gene Cluster C by red dots. **(C)** Kaplan-Meier curve presents the survival of patients with high and low TMB values, designated as H-TMB and L-TMB, respectively. **(D)** Survival analysis for patients grouped by both CXCL score and TMB values. **(E,F)** Waterfall plot displays the distribution of somatic mutations in patients with high **(E)** and low **(F)** CXCL scores. Each column represents an individual patient.

High levels of tumor mutational burden (TMB) and the presence of cancer gene mutations have been positively linked to sensitivity to immunotherapy in some types of tumors (Bai et al., 2020). To assess the immunotherapeutic response of individual OC patients, we analyzed the relationship between the CXCL score and TMB. Although there was not a strong correlation between TMB and CXCL score, there was a trend towards patients with high CXCL scores having higher TMB values (Figures 6A, B). We then divided patients into two classes based on their TMB value, with 108 patients in the high TMB (H-TMB) class and 145 patients in the low TMB value class (L-TMB). Survival analysis showed that patients in the H-TMB group had better survival outcomes than those in the L-TMB group (*p* < 0.001) (Figure 6C). The results of the joint analysis of the CXCL score and TMB showed that patients in the H-TMB group with high CXCL scores had the best survival, while those in the L-TMB group with low CXCL scores had the worst outcome (Figure 6D). We also analyzed the somatic mutations of patients in the two CXCL score groups using the TCGA-OC cohort. Results showed that the somatic mutation rate of patients in the high CXCL score group (98.72%) was higher than in the low CXCL score group (96%). The mutation rate of the TP53 gene was 91% in the high CXCL score group and 83% in the low CXCL score group (Figures 6E, F). Collectively, these results suggest that higher CXCL scores are associated with higher TMB values, indicating better responses to immunotherapy.

### Predictive value of CXCL score for immunotherapy outcomes

**FIGURE 7:**
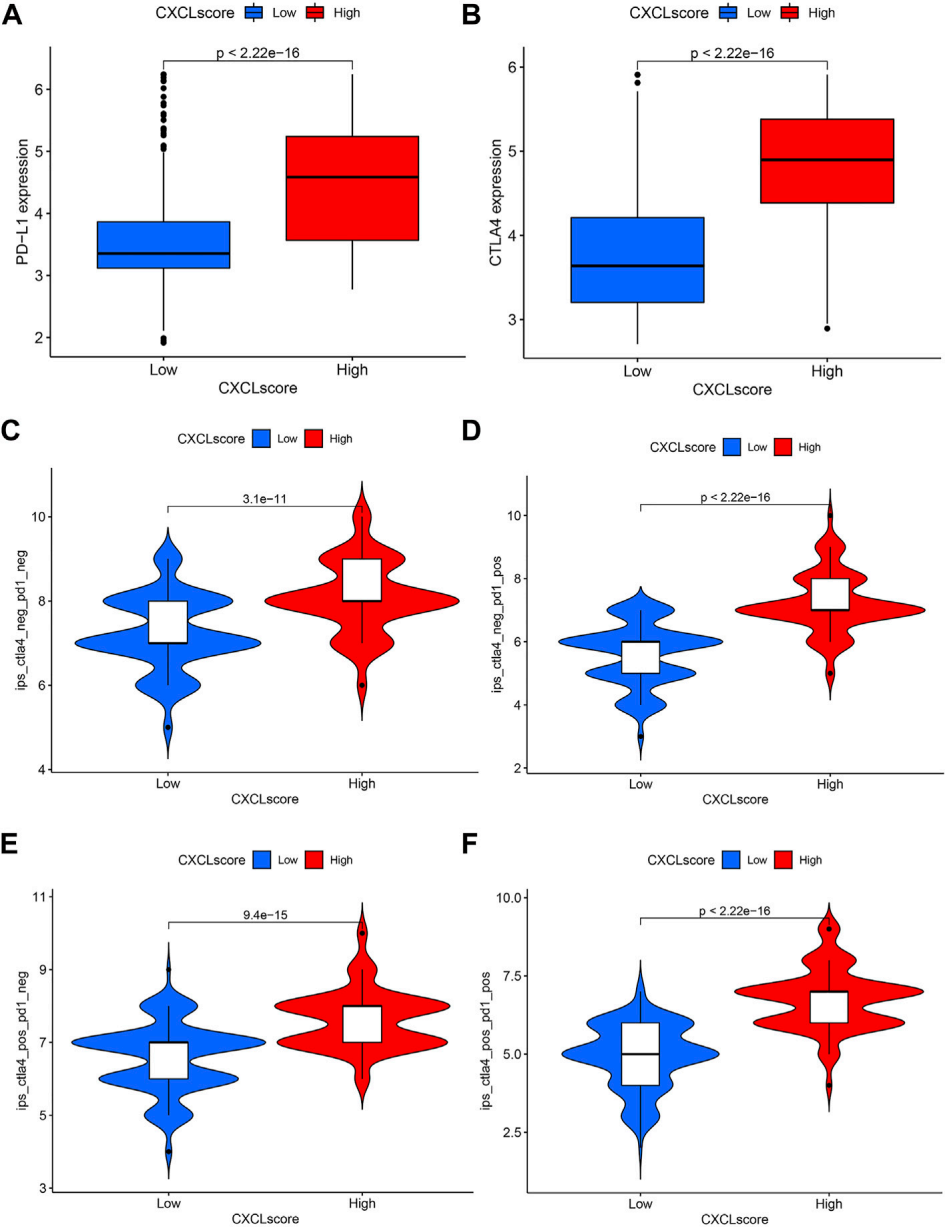
The predictive value of the CXCL score in immunotherapy. **(A,B)** Box plots present the expression levels of PD-L1 **(A)** and CTLA4 **(B)** in patients with high and low CXCL scores. **(C–F)** Violin plots display the IPS scores of patients with low and high CXCL scores who received non-ICI therapy **(C)**, PD-1 therapy alone **(D)**, CTLA4 therapy alone **(E)**, or a combination of PD-1 and CTLA4 therapy **(F)**.

Expression of PD-L1 is a clinically recognized indicator for anti-PD-1/PD-L1 therapy in cancer patients (Luchini et al., 2019; Twomey and Zhang, 2021), while CTLA-4 is another potential target for immune checkpoint inhibitor (ICI) therapy (Rowshanravan et al., 2018). To determine the predictive value of the CXCL score in immunotherapy, we compared the expression levels of PD-L1 and CTLA-4 between patients with high and low CXCL scores. Results showed that patients with high CXCL scores expressed higher levels of PD-L1 (Figure 7A) and CTLA-4 (Figure 7B) compared to those with low CXCL scores, indicating that these patients may benefit more from ICI treatment.

Additionally, the immunophenoscore (IPS) has been found to predict the efficacy of anti-PD-1 and anti-CTLA-4 therapy (Charoentong et al., 2017). To further validate the value of the CXCL score in predicting immunotherapy response, we evaluated the sensitivity of ICI therapy using IPS. Results indicated that patients with high CXCL scores had better survival outcomes compared to those with low CXCL scores with either anti-PD-1 or anti-CTLA-4 therapy (Figures 7C–E). Clinical trials have shown that combination therapy with anti-PD-1 and anti-CTLA-4 is effective in treating lung cancer and melanoma (Wolchok et al., 2013; Hellmann et al., 2019). The results as depicted in Figure 7F indicate that patients with high CXCL scores have higher IPS levels compared to those with low CXCL scores when treated with the combination therapy of anti-PD-1 and anti-CTLA-4 (Figure 7F). This suggests that patients with high CXCL scores may experience a greater benefit from this combination therapy.

Taken together, the CXCL score may serve as a positive predictor for a patient’s response to immunotherapy and could be used to select the appropriate patient population for the treatment in ovarian cancer.

## Discussion

The CXCL family genes play a crucial role in the progression of tumors and in the microenvironment (Bikfalvi and Billottet, 2020). However, the full extent of their importance in OC remains unclear. Our study confirmed the expression of CXCLs in OC tissue compared to normal tissue and found that most CXCLs were overexpressed in OC tissue. Based on this expression profile, we were able to group OC patients into three CXCL clusters. Of these clusters, patients in cluster C had better survival rates and higher infiltration of immune cells. By using DEGs among CXCL clusters, we identified three gene clusters, and developed a CXCL score to predict prognosis and immunotherapy response in individual patients. The relationships of the CXCL score with clinical outcomes, cell infiltration levels, somatic mutations, and immunotherapy sensitivity were also studied to evaluate the value of CXCLs in OC.

The CXCLs are involved in tumor progression. For instance, CXCL1 and CXCL8 have been found to stimulate ovarian cancer cell growth *via* activation of the p38 and Wnt/β-catenin pathway (Duckworth et al., 2016; Wen et al., 2020; Park et al., 2021). CXCL5, secreted by ovarian cancer-associated mesothelial cells, has been demonstrated to have tumor-promoting properties (Peng et al., 2019). Furthermore, high levels of CXCL11 expressed in cancer-associated fibroblasts in ovarian cancer biopsies were found to facilitate cancer cell metastasis (Lau et al., 2014). Our results align with these findings and show that CXCL1, CXCL5, CXCL8, and CXCL11 are overexpressed in ovarian cancer. Additionally, previous studies have found that overexpressed CXCL9, CXCL10, and CXCL13 are positively associated with better overall survival, while elevated CXCL12 and CXCL14 levels are linked to poor outcomes (Popple et al., 2012; Bronger et al., 2016; Li et al., 2020; Ukita et al., 2022). Our study validated these findings and showed that CXCL9, CXCL10, CXCL12, CXCL13, and CXCL14 are independent risk factors for clinical outcomes. High levels of CXCL9, CXCL10, and CXCL13 were found to be associated with good prognosis, while high expression levels of CXCL12 and CXCL14 were correlated with poor survival. These findings suggest that CXCLs may play a crucial role in the progression of ovarian cancer.

The relationship between genomic profiling and survival outcome in cancer patients has gained significant attention in recent years. Research has shown that chemokine ligands CXCL10 and CXCL11 have anti-angiogenic properties and can effectively inhibit tumor progression (Romagnani et al., 2004; Billottet et al., 2013). Additionally, CXCL9, CXCL10, and CXCL13 are involved in attracting CD8 effector T cells, and a high infiltration of T lymphocytes has been linked to improved survival outcomes (Sato et al., 2005; Harlin et al., 2009; Ukita et al., 2022). Our study found three subfamilies of OC patients with distinct survival outcomes based on the expression of the CXCLs. Patients in cluster C with the highest expression of CXCL9/10/11/13, who also showed activation of immune-related pathways and high infiltration of immune cells including T cells, had the best survival outcomes. Conversely, patients in cluster B, characterized by low expression of CXCL9/10/11/13 and low immune cell infiltration, had a poor prognosis. These findings highlight the potential use of CXCL expression as a biomarker for the treatment and prognosis of ovarian cancer.

Immunotherapy, including anti-PD-1/PD-L1 and CTLA-4, is commonly employed in various solid tumors, but its efficacy in OC is limited (Marabelle et al., 2020; Robert, 2020; Marcus et al., 2021; O'Malley et al., 2022). Identifying reliable biomarkers to direct immunotherapy may broaden the reach of immunotherapy to OC patients (Zamarin and Jazaeri, 2016). In our study, we utilized the CXCL score as a biomarker to predict individual patients’ responses to immunotherapy in OC. High PD-L1/CTLA4 expression levels, high IPS scores, and a greater number of cancer gene mutations have been established as solid predictive biomarkers for patients who are more likely to benefit from immunotherapy (Egen et al., 2002; Garon et al., 2015; Herbst et al., 2016; Charoentong et al., 2017; Bai et al., 2020; Marabelle et al., 2020). We examined the prognostic significance of the CXCL score in immunotherapy response by evaluating these predictive biomarkers. Our findings indicate that patients with high CXCL scores had elevated levels of PD-L1/CTLA4, high IPS scores, and a high frequency of cancer gene mutations. Therefore, the CXCL score can be used as a predictor of immunotherapy response in OC patients.

Immune cells express chemokine receptors and can be attracted to tumors through chemokines, including CXCLs. Our study revealed a positive correlation between the CXCL score and the infiltration of immune cells. However, the underlying mechanism behind this connection is still unclear. It is plausible that the CXCL score serves as a measure of the concentration of immune cells in the tumor microenvironment. A high CXCL score, indicating a high expression of anti-tumor CXCL, may attract a larger number of immune cells to the tumor, resulting in a more inflamed microenvironment and enhanced response to immunotherapy. On the other hand, a low CXCL score may indicate a lack of immune cell infiltration and a less favorable tumor microenvironment, leading to a weaker response to immunotherapy. Further studies are needed to fully understand the mechanisms linking the CXCL score to clinical outcomes and immunotherapy responses in patients.

In conclusion, our comprehensive evaluation of CXCLs in ovarian cancer has uncovered a promising biomarker that could forecast the prognosis and response to immunotherapy for individual patients. This has the potential to enhance the implementation of precision-targeted, personalized immunotherapy in ovarian cancer patients.

## Data Availability

Publicly available datasets were analyzed in this study. This data can be found here: The Cancer Genome Atlas (TCGA) (https://portal.gdc.cancer.gov/) ,the UCSC Xena database (https://xena.ucsc.edu/),and Gene Expression Omnibus (GEO) (https://www.ncbi.nlm.nih.gov/geo/) databases.
