# Supplementary material for: Comprehensive bioinformatic analysis constructs a CXCL model for predicting survival and immunotherapy effectiveness in ovarian cancer
Source: Front Pharmacol. 2023 Mar 9;14:1127557. doi: 10.3389/fphar.2023.1127557 (PMC10034089; doi:10.3389/fphar.2023.1127557)
Supplement: Supplementary file 1 [file DataSheet1.pdf]

Supplementary Material

Comprehensive bioinformatic analysis constructs a CXCL model for predicting survival and immunotherapy effectiveness in ovarian cancer

Shuang Li[1,3], Dawei Zou[2]* and Zhaoqian Liu[1,3]*

[1]Department of Clinical Pharmacology, Hunan Key Laboratory of Pharmacogenetics, and National Clinical Research Center for Geriatric Disorders, Xiangya Hospital, Central South University, Changsha 410008, P. R. China

[2]Immunobiology & Transplant Science Center, Department of Surgery, Houston Methodist Research Institute & Institute for Academic Medicine, Houston Methodist Hospital, Houston, TX 77030, USA

[3]Institute of Clinical Pharmacology, Central South University, Changsha 410078, P. R. China.

[*]**Corresponding author:**

Zhaoqian Liu. Department of Clinical Pharmacology, Hunan Key Laboratory of Pharmacogenetics, Xiangya Hospital, Central South University, Changsha 410008, P. R. China. Tel: +86 731 89753845, Fax: +86 731 82354476, E-mail: zqliu@csu.edu.cn.

Dawei Zou. Department of Surgery, Houston Methodist Research Institute & Institute for Academic Medicine, Houston, TX 77030, USA. Tel: +1 346 719 8497, E-mail: dzou@houstonmethodist.org.

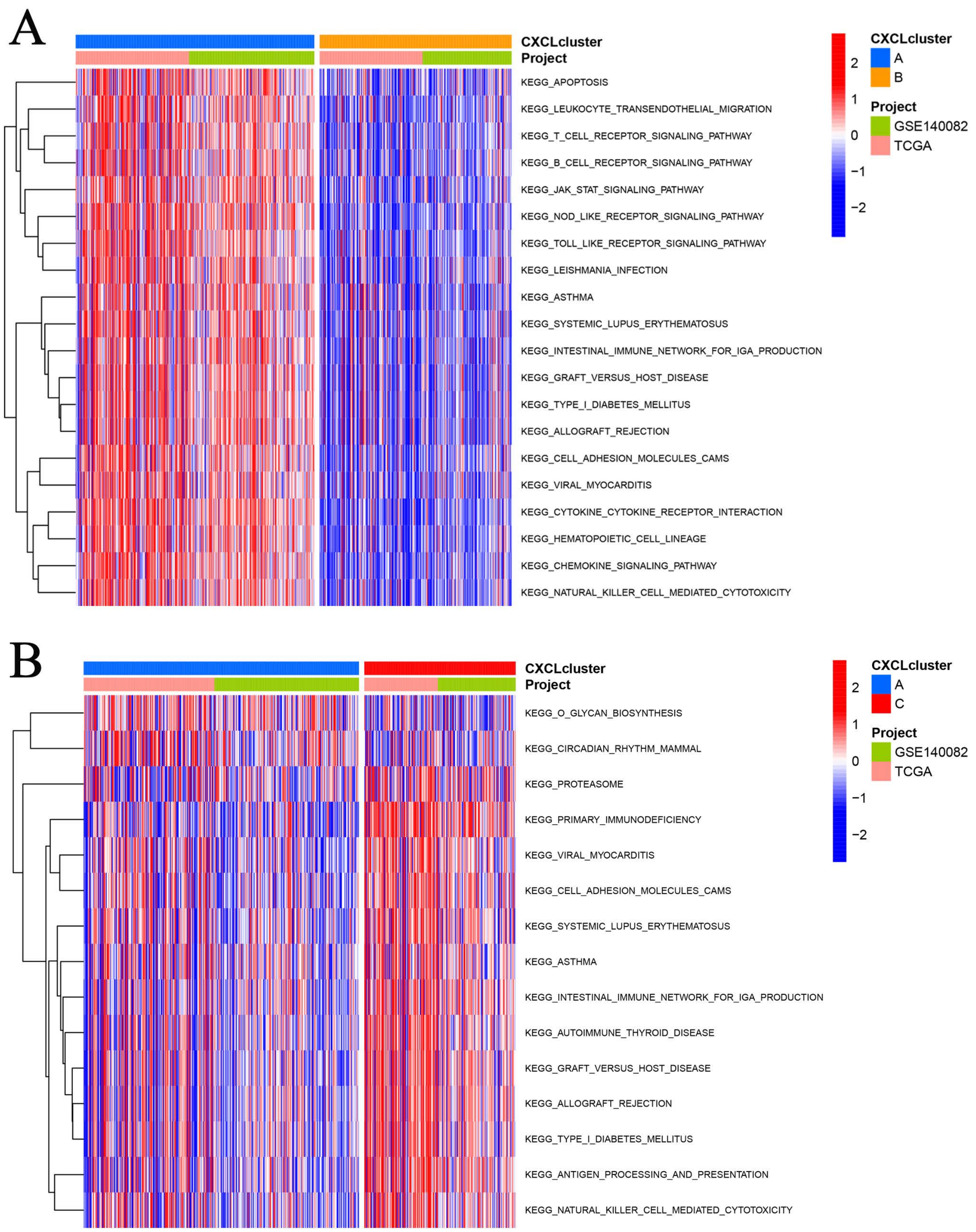

Supplementary figure 1 Heatmap represents the results of KEGG pathway enrichment analysis between CXCL cluster B and C (**A**) or between CXCL cluster A and C (**B**).

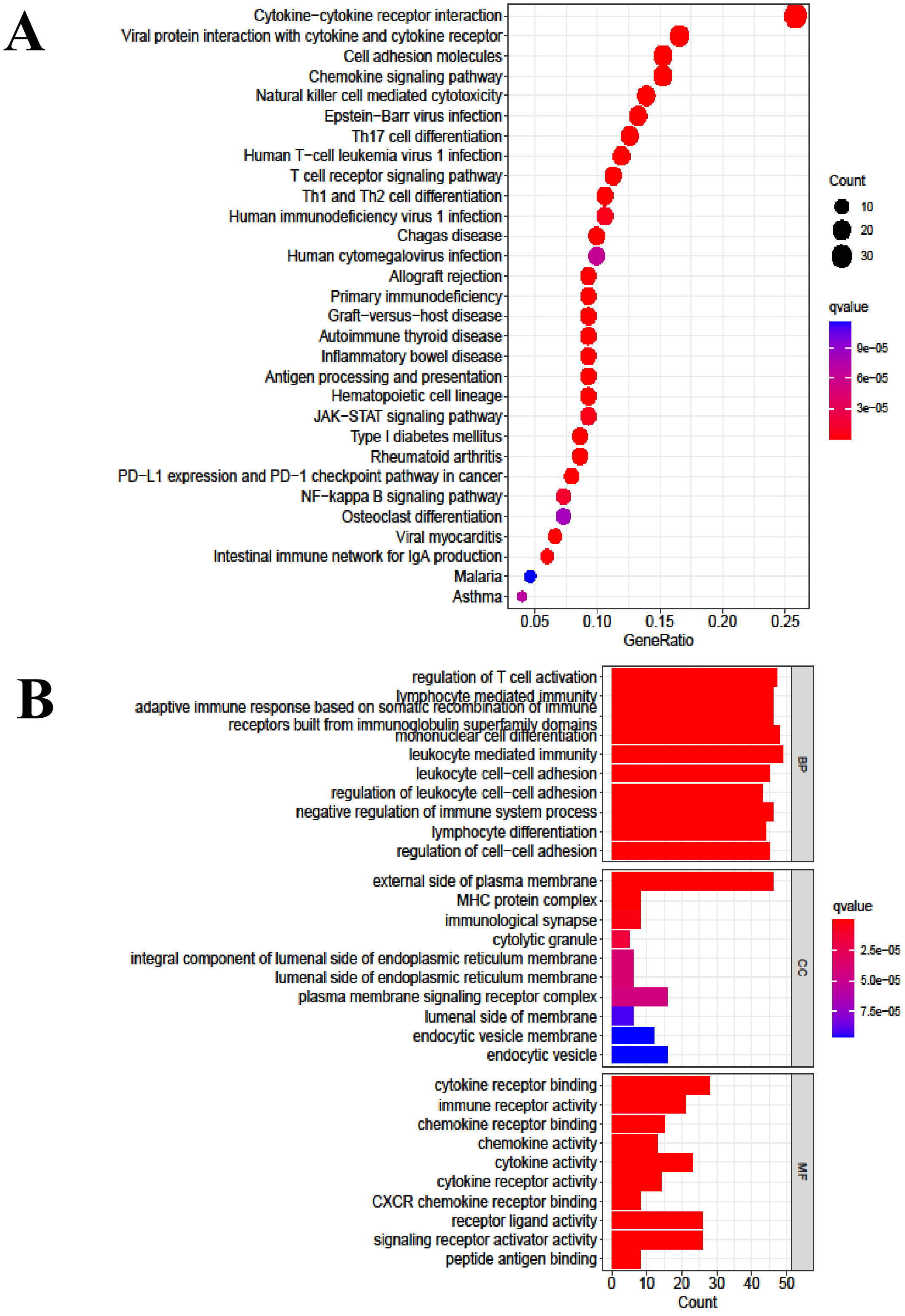

Supplementary figure 2 GSVA enrichment analysis based on 244 shared DEGs. (**A**) Dot plots represent 30 KEGG enrichment pathways. (**B**) Box plots display the top 10 GO terms in each biological process.

A

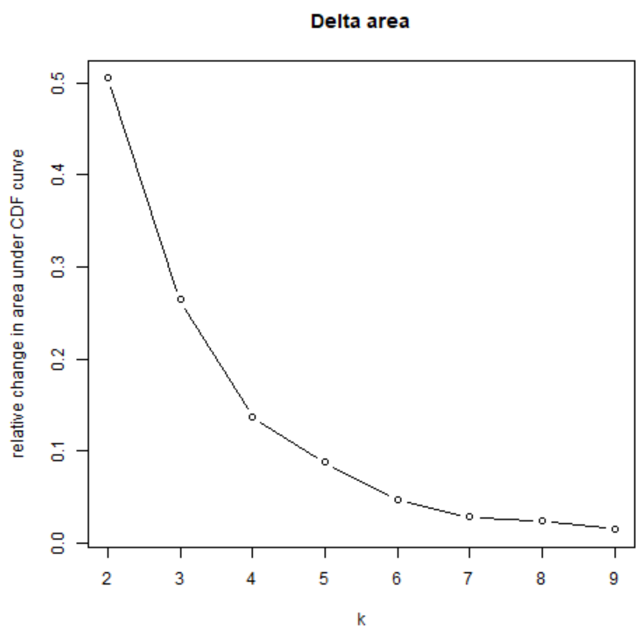

B

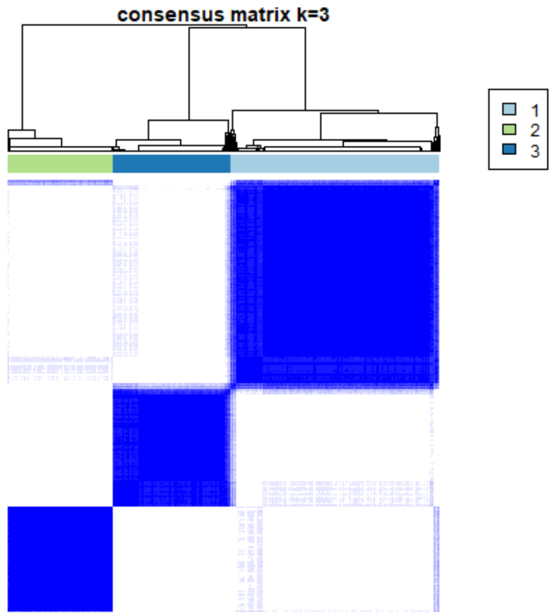

Supplementary figure 3 Consensus clustering analysis based on OS-related shared DEGs of CXCL clusters. (**A**)Consensus CDF from k =2 to 9. (**B**) Heatmap of the consensus matrix (k = 3).

Table S1. Univariate COX analysis and KM analysis show the prognostic value of 16 CXCLs in OC.

ID	HR	HR.95L	HR.95H	*p*-value	km
CXCL1	0.997123	0.93324	1.065378	0.932031	0.082844
CXCL2	0.949344	0.883321	1.020303	0.157521	0.027271
CXCL3	0.95476	0.842333	1.082192	0.46891	0.172617
CXCL4	0.946135	0.785129	1.140157	0.56071	0.046224
CXCL5	0.998051	0.913915	1.089933	0.965374	0.04508
CXCL6	1.020485	0.937641	1.110648	0.638772	0.023663
CXCL7	1.100093	0.968335	1.249778	0.142756	0.024492
CXCL8	0.971027	0.902014	1.045321	0.434439	0.039965
CXCL9	0.895328	0.841657	0.952421	0.000456	2.25E-05
CXCL10	0.917978	0.870476	0.968071	0.001594	1.31E-07
CXCL11	0.860153	0.794843	0.930828	0.000185	3.58E-08
CXCL12	1.062638	0.981835	1.150091	0.132157	0.004161
CXCL13	0.844875	0.785938	0.908232	4.90E-06	1.21E-06
CXCL14	1.049345	0.993994	1.107777	0.081492	0.001643
CXCL16	0.983203	0.857066	1.127903	0.808923	0.151989
CXCL17	0.994427	0.948267	1.042835	0.817753	0.341343

Table S2. COX and KM survival analysis show the prognostic value of 94 OS-related shared DEGs in OC.

ID	HR	HR.95L	HR.95H	pvalue
CCL5	0.896529	0.828726	0.969879	0.006485
CXCL10	0.917978	0.870476	0.968071	0.001594
IL2RG	0.890307	0.818828	0.968025	0.006508
TNFSF13B	0.900052	0.813568	0.995729	0.041051
GZMB	0.902196	0.842271	0.966383	0.003334
CH25H	1.145363	1.049545	1.249929	0.002328
GJB2	1.08477	1.020107	1.153533	0.009465
CD3E	0.906137	0.828354	0.991224	0.031361
CXCL11	0.860153	0.794843	0.930828	0.000185
P2RY10	0.818149	0.715439	0.935605	0.003363
IL21R	0.872076	0.765396	0.993625	0.039779

**LTA**	**0.801501**	**0.706924**	**0.90873**	**0.000553**
**GBP1**	**0.884256**	**0.806842**	**0.969097**	**0.008501**
**LRRC15**	**1.083855**	**1.012966**	**1.159705**	**0.019635**
**CTHRC1**	**1.126541**	**1.03649**	**1.224415**	**0.005061**
**CXCL13**	**0.844875**	**0.785938**	**0.908232**	**4.90E-06**
**CD2**	**0.881638**	**0.81673**	**0.951704**	**0.001244**
**TREM1**	**1.19916**	**1.045301**	**1.375665**	**0.009533**
**AIM2**	**0.871099**	**0.769081**	**0.98665**	**0.029898**
**SH2D1A**	**0.77806**	**0.664899**	**0.910481**	**0.001751**
**SLAMF6**	**0.878813**	**0.795364**	**0.971018**	**0.011158**
**NKG7**	**0.935828**	**0.878642**	**0.996736**	**0.039247**
**ITGBL1**	**1.167906**	**1.039882**	**1.31169**	**0.008789**
**CD3D**	**0.894195**	**0.831535**	**0.961578**	**0.002553**
**TIGIT**	**0.77609**	**0.640934**	**0.939747**	**0.009417**

**ADAMDEC1**	**0.901914**	**0.828978**	**0.981268**	**0.016418**
**ICOS**	**0.841058**	**0.758851**	**0.93217**	**0.000972**
**CTLA4**	**0.815784**	**0.712794**	**0.933655**	**0.003107**
**HLA-B**	**0.873416**	**0.780515**	**0.977375**	**0.018334**
**GZMA**	**0.924006**	**0.867845**	**0.983802**	**0.013496**
**GBP4**	**0.883315**	**0.814894**	**0.957481**	**0.002559**
**CD3G**	**0.820173**	**0.739675**	**0.909432**	**0.000169**
**FBN1**	**1.136584**	**1.040825**	**1.241153**	**0.004358**
**ANKRD22**	**0.855521**	**0.744349**	**0.983296**	**0.02801**
**GZMK**	**0.883165**	**0.792167**	**0.984616**	**0.02513**
**ITK**	**0.899838**	**0.826827**	**0.979296**	**0.014504**
**GBP5**	**0.883189**	**0.823762**	**0.946903**	**0.000474**
**FIBIN**	**1.146438**	**1.0253**	**1.281889**	**0.016464**
**HLA-A**	**0.856447**	**0.744471**	**0.985265**	**0.030189**

**GPR171**	**0.800759**	**0.678589**	**0.944923**	**0.008521**
**TBX21**	**0.757514**	**0.580232**	**0.988964**	**0.041197**
**PYHIN1**	**0.825012**	**0.718155**	**0.947769**	**0.006569**
**CCL19**	**0.9299**	**0.87999**	**0.982642**	**0.009821**
**HLA-F**	**0.885053**	**0.804675**	**0.97346**	**0.011948**
**IRF4**	**0.825944**	**0.712932**	**0.956871**	**0.010859**
**SIT1**	**0.870221**	**0.772694**	**0.980057**	**0.021898**
**IDO1**	**0.915329**	**0.860102**	**0.974102**	**0.00533**
**KLRB1**	**0.847472**	**0.765144**	**0.938659**	**0.001503**
**CXCL9**	**0.895328**	**0.841657**	**0.952421**	**0.000456**
**CXCR6**	**0.916945**	**0.854362**	**0.984112**	**0.016217**
**CD38**	**0.829461**	**0.760567**	**0.904595**	**2.38E-05**
**CCR7**	**0.903157**	**0.839958**	**0.971112**	**0.005924**
**FCRL3**	**0.746602**	**0.606524**	**0.91903**	**0.005844**

**CD79A**	**0.886653**	**0.813704**	**0.966141**	**0.006027**
**FCRL5**	**0.81268**	**0.671362**	**0.983746**	**0.033329**
**PSMB9**	**0.88437**	**0.814524**	**0.960205**	**0.003418**
**LCK**	**0.847107**	**0.728967**	**0.984394**	**0.030371**
**UBD**	**0.889244**	**0.809553**	**0.97678**	**0.01427**
**LY9**	**0.784453**	**0.642118**	**0.95834**	**0.017476**
**TAP1**	**0.853726**	**0.776829**	**0.938235**	**0.001024**
**CD27**	**0.900156**	**0.820722**	**0.987278**	**0.025641**
**UBE2L6**	**0.852117**	**0.74737**	**0.971546**	**0.016788**
**APOBEC3G**	**0.860545**	**0.766474**	**0.966162**	**0.010997**
**CCL13**	**0.885745**	**0.819028**	**0.957897**	**0.002393**
**IL18BP**	**0.841052**	**0.722059**	**0.979655**	**0.026145**
**CD96**	**0.859873**	**0.770062**	**0.960159**	**0.007311**
**CLEC12A**	**0.87147**	**0.772621**	**0.982966**	**0.025113**

**ETV7**	**0.891643**	**0.810527**	**0.980878**	**0.018439**
**TAP2**	**0.853289**	**0.758322**	**0.96015**	**0.008402**
**STAT1**	**0.79335**	**0.703903**	**0.894164**	**0.000149**
**IGLL1**	**0.833755**	**0.725109**	**0.95868**	**0.0107**
**SLA2**	**0.868161**	**0.76311**	**0.987674**	**0.031678**
**SP140**	**0.860129**	**0.759948**	**0.973515**	**0.017088**
**TNFRSF13B**	**0.892288**	**0.808727**	**0.984481**	**0.023103**
**CD40LG**	**0.724545**	**0.588959**	**0.891344**	**0.002303**
**STAT4**	**0.843472**	**0.749156**	**0.949661**	**0.004898**
**LAX1**	**0.85449**	**0.767996**	**0.950725**	**0.003877**
**ZBP1**	**0.915269**	**0.849815**	**0.985765**	**0.019352**
**SLAMF1**	**0.77352**	**0.662615**	**0.902986**	**0.001144**
**PLA2G2D**	**0.822484**	**0.704403**	**0.960359**	**0.013455**
**UBASH3A**	**0.790653**	**0.657382**	**0.950941**	**0.012629**

**IL21**	**0.602966**	**0.412618**	**0.881123**	**0.008952**
**STAP1**	**0.786228**	**0.64692**	**0.955534**	**0.015645**
**BATF2**	**0.896953**	**0.824074**	**0.976276**	**0.011894**
**FCRLA**	**0.885741**	**0.799721**	**0.981013**	**0.019927**
**DERL3**	**0.875973**	**0.794724**	**0.965529**	**0.007669**
**GPR18**	**0.888967**	**0.798618**	**0.989537**	**0.031373**
**CD72**	**0.863019**	**0.761447**	**0.978141**	**0.021116**
**CD274**	**0.846413**	**0.749242**	**0.956186**	**0.007361**
**BTN3A2**	**0.898746**	**0.812624**	**0.993996**	**0.037789**
**ICAM3**	**0.85412**	**0.734208**	**0.993616**	**0.041059**
**AMPD1**	**0.877646**	**0.77113**	**0.998875**	**0.048039**
**HLA-DOB**	**0.833904**	**0.770401**	**0.902643**	**6.97E-06**
**APOL4**	**0.897624**	**0.810777**	**0.993773**	**0.037501**
